# Lack of Association between Hepatitis C Virus *core* Gene Variation 70/91aa and Insulin Resistance

**DOI:** 10.3390/ijms18071444

**Published:** 2017-07-21

**Authors:** Letícia de Paula Scalioni, Allan Peres da Silva, Juliana Custódio Miguel, Márcia Paschoal do Espírito Santo, Vanessa Alves Marques, Carlos Eduardo Brandão-Mello, Cristiane Alves Villela-Nogueira, Lia Laura Lewis-Ximenez, Elisabeth Lampe, Livia Melo Villar

**Affiliations:** 1 Livia Melo Villar, Viral Hepatitis Laboratory, Helio and Peggy Pereira Pavilion, Ground Floor, Room B09, FIOCRUZ Av. Brasil, 4365-Manguinhos, Rio de Janeiro, RJ 210360-040, Brazil; le_scali@hotmail.com (L.d.P.S.); allan@ioc.fiocruz.br (A.P.d.S.); julicm@ioc.fiocruz.br (J.C.M.); paschoalms@gmail.com (M.P.d.E.S.); vmarques@ioc.fiocruz.br (V.A.M.); llewis@ioc.fiocruz.br (L.L.L.-X.); elampe@ioc.fiocruz.br (E.L.); 2 Gaffrée & Guinle University Hospital, Federal University of Rio de Janeiro State, Rio de Janeiro, RJ 20270-001, Brazil; cedubrandao@gmail.com; 3 University Hospital Clementino Fraga Filho, School of Medicine, Federal University of Rio de Janeiro, Rio de Janeiro, RJ 21941-913, Brazil; crisvillelanog@gmail.com

**Keywords:** amino acid, core mutation, insulin resistance, hepatitis C

## Abstract

The role of hepatitis C virus (HCV) in insulin resistance (IR) is not fully understood. The aim of this study was to determine the impact of amino acid (aa) substitutions in the *core* region of HCV according to IR and to identify clinical and laboratory associations. Ninety-two treatment-naive HCV patients were recruited to determine laboratory data and blood cell count. IR was determined using Homeostasis Model Assessment (HOMA) index where IR was defined as HOMA ≥2. HCV RNA load and genotype were determined by Abbott Real time HCV. HCV *core* region was determined by direct nucleotide sequencing. Bivariate analysis was conducted using HOMA IR ≥2 as a dependent factor. IR prevalence was 43.5% (*n* = 40), vitamin D sufficiency was found in 76.1% (*n* = 70) and 72.8% (*n* = 67) had advanced liver fibrosis. In the bivariate analyses, elevated values of γGT (*p* = 0.024) and fibrosis staging (*p* = 0.004) were associated with IR, but IR was not related to core mutations. The presence of glutamine in position 70 was associated with low vitamin D concentration (*p* = 0.005). In the multivariate analysis, no variable was independently associated with HOMA-IR. In conclusion, lack of association between IR and HCV core mutations in positions 70 and 91 suggests that genetic variability of this region has little impact on IR.

## 1. Introduction

Hepatitis C virus (HCV) infection is a serious health problem affecting over 170 million people worldwide [1]. Chronic hepatitis C (CHC) is associated with many extrahepatic manifestations that contribute to morbidity and mortality [2].

CHC when associated with metabolic diseases may lead to rapid progression of the disease, increasing the risk of developing hepatocellular carcinoma (HCC) and advanced fibrosis [3]. These data become extremely relevant due to the high prevalence of obesity and metabolic syndromes observed worldwide [3]. 

Further studies are needed to address the various metabolic manifestations possible in patients with CHC. Currently available antiviral treatment for HCV has been shown to be quite effective (>90%), but it is important to recognize and identify irreversible and associated metabolic damage, thereby reducing the morbidity and mortality associated with HCV [3].

Some studies have shown that CHC is associated with insulin resistance (IR) and DM2 (diabetes mellitus type 2) [4,5], which is characterized by hyperinsulinemia in patients with normal fasting blood glucose and with an increased risk of developing diabetes mellitus (T2DM), heart disease, and nonalcoholic fatty liver disease [6,7]. 

Hepatitis C virus promotes the development of IR and DM2 by increasing the inflammatory response, such as high production of interleukin 6 (IL-6), tumor necrosis factor (TNF), and oxidative stress, leading to interference in the insulin signaling pathway in hepatocytes [8].

Previous studies provided a direct experimental evidence for the contribution of HCV core protein in the development of insulin resistance (IR), but the clinical impact of HCV core region on IR is still not clear [8,9]. Akuta et al. [9] demonstrated that patients infected with HCV genotype 1b and who had 70Q core mutation had higher rates of IR than patients with HCV genotype 1b without the mutation, indicating that this mutation is associated with the development of IR. In addition, it has been suggested that core 70Q mutation is associated with a higher incidence of HCC and mutations in 70 and/or 91 positions are important predictors of IR in patients without cirrhosis or DM [10,11].

Despite this evidence, there are few studies on the genomic variability of the HCV 70/91 aa relating to the development of IR [8,9,10]. The aim of this study is to determine the impact of aa substitutions in the *core* region of HCV with insulin resistance and to identify associations with clinical and laboratory data. 

## 2. Results

### 2.1. Clinical Characteristics

The baseline characteristics of 92 patients with CHC are shown in Table 1. There were 55 women and 37 men, with a mean age of 54.84 (±10.88) years. Elevated mean values of ALT (73.51 ± 58.76 IU/mL), AST (72.15 ± 44.04 IU/mL), alkaline phosphatase (136.21 ± 75.66 IU/mL), and γGT (94.25 ± 77.66 IU/mL) were observed in patients. HOMA index mean was also higher than the cutoff established in this study (3.08 ± 2.74), where 43.5% (*n* = 40) presented IR. Lipids mean values were classified as normal as well as blood glucose value (92.84 ± 13.51 ng/mL), platelets (187.13 ± 72.72 10^3^/mm), and hemoglobin (14.04 ± 1.27 ng/mL) (anemic classification: hemoglobin values <12 ng/mL for women and <13 ng/mL for men). Using the cutoff value of 20 ng/mL for hypovitaminosis, most individuals had adequate vitamin D concentrations (76.1%, *n* = 70). Genotype 1b was the most prevalent 64.1% (*n* = 59), followed by genotype 1a, 25% (*n* = 23) and genotype 3, 10.9% (*n* = 10). HCV RNA log median was high, 5.81 UI/mL (1.86–7.49). After inclusion in the study, most individuals were not treated (56.5%) while 40 individuals were treated after of which half of them achieved sustained viral response (SVR). Using fibrosis algorithm, 72.8% (*n* = 67) presented advanced fibrosis.

### 2.2. Insulin Resistance and Associated Factors

To assess the influence of IR on HCV infection, comparative analysis of demographic and clinical variables was evaluated according to previously defined IR cutoff values (HOMA ≥ 2). Elevated values of γGT (*p* = 0.024) and advanced fibrosis (*p* = 0.004) were both associated with HOMA ≥ 2 in CHC patients in the univariate analysis (Table 2), but associations were lost when using the multivariate analysis. We made an analysis using HOMA >3.0 and 3.5, but no variable was statistically significant. 

### 2.3. Substitution of Hepatitis C core Amino Acids 70 and 91 versus Insulin Resistance Development and Laboratorial Data

IR was evaluated regarding the presence or absence of mutations at *core* positions 70 and 91. In this study, R70Q (Q = 26; H = 2) and M91L/C (L = 12; C = 2) *core* substitutions were present exclusively in HCV genotype 1b; while genotype 1a had R70W/P/Q (W = 1; P = 1; Q = 1) and cysteine in all sequences at position 91 and genotype 3, the mutations P70Q/R (Q = 4; R = 10) and C91L (*n* = 1). A total of 92 subjects were studied, where 52 presented insulin resistance with HOMA-IR ≥ 2. There was no statistical association between the presence of IR and mutations in the *core* region (Table 3). Analysis using HOMA >3.0 and 3.5 was carried out, but no variable was statistically significant (data not shown). 

**Figure 1:**
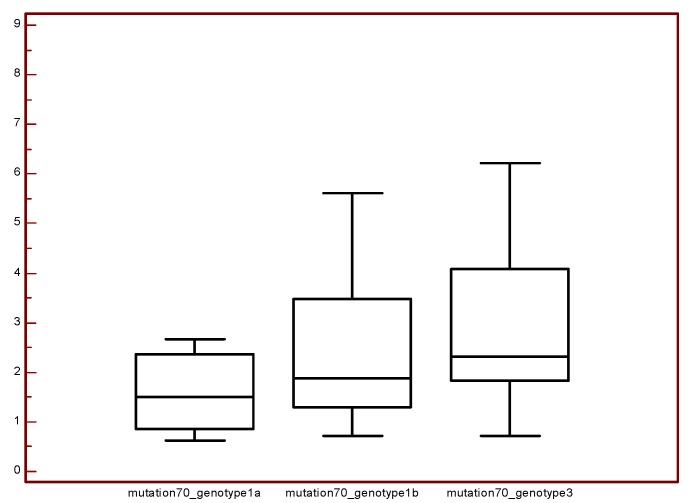
Boxplots for each genotype and mutation 70 according to HOMA values for each patient. Mutation70_genotype1a: Median, 1.501; Minimum, 0.6321; Maximum, 2.669. Mutation70_genotype1b: Median, 1.881; Minimum, 0.7246; Maximum, 6.793. Mutation70_genotype3: Median, 2.312; Minimum, 0.7104; Maximum, 8.880.

Besides, all mutations were analyzed as dependent variables in a univariate model with biochemical, virological, and demographic data (data not shown). The presence of amino acid glutamine (Q) in position 70, was associated with lower 25(OH)D concentration (*p* = 0.005) (CI: 0.09091–0.4789). When protein sequences were evaluated in VESPA software, no variable position (signature) was found in the presence of IR. A boxplot was designed for each genotype and mutation core aa 70 according to HOMA values. Median HOMA values were higher in genotype 3a samples with any mutation in position 70 (Figure 1).

## 3. Discussion

The present study did not identify an association between insulin resistance and core amino acid 70 and 91 substitutions. Conversely, *core* mutations R70Q and M91L/C had been previously reported to be associated with IR in genotype 1b [9]. In addition, these mutations had been related to: increased HCC risk, [12,13], non-virological response to interferon-based therapy in Japanese patients [10], variable responses to double (IFN/RBV) [14,15], and triple therapy (telaprevir plus INF/RBV) [16,17], and liver steatosis [18]. On the other hand, some studies showed that HCV core protein increases IRS-1 phosphorylation, modulates Forkhead box transcriptional regulators (FoxO1 and FoxO2), and activates the mTOR/S6K1 pathway that could contribute to promote IR. These data show that HCV core protein have an important role in developing IR even in the absence of core mutations [19,20,21]. 

The lack of association of these mutations in the present study could be due to the prevalence of obese patients in Brazil compared to Japan that could interfere in the prevalence of IR. The standard definition of overweight is BMI 25.0–29.9 kg/m^2^, and obesity, BMI ≥ 30 kg/m^2^. These cut-offs are based on studies involving Western populations. According to this cutoff, the prevalence of obesity (≥30) is only 2–4% in Asian populations [22], while in young Brazilian adults the prevalence of obesity is 8.1% (males) and 10.7% (females) [23]. In addition, only genotype 1b patients were included in the Japanese cohort while in this study, genotypes 1a, 1b, and 3 were included that may have contributed to the differences in results. 

The IR prevalence was 42.5%, similar to previous studies (44.1%) in HCV patients [24]. IR was associated with elevated γGT levels and fibrosis as previously shown in HCV-monoinfected patients [25]. Everhart and collaborators [25] evaluated high serum γGT levels as a predictor for virological response and clinical end points in 1319 patients with significant liver fibrosis included in the hepatitis C anti-viral treatment against cirrhosis (HALT-C) trial. 

The presence of core amino acid Q in position 70 was associated with low concentrations of vitamin D. In obese subjects, the endocrine system with insufficient vitamin D concentration is characterized by high PTH level and 1,25(OH)_2_D_3_ due to the negative feedback by the low hepatic synthesis of 25(OH)D, which also contributes to a higher concentration of intracellular calcium, thus decreasing secretion and sensitivity to insulin [26].

Vitamin D can affect insulin response to glucose directly or indirectly [27]. The direct effect seems to be mediated by the binding 1,25(OH)_2_D_3_ with a vitamin D receptor in the β-cell. Alternatively, the activation of vitamin D may occur inside the β-cells by 1α-hydroxylase expressed into these cells [28]. After the relationship between the metabolic pathways of vitamin D and insulin has been established, *core* protein may be a key part of this not well-established cascade. 

This study presents some limitations as the small number of treated patients, lack of information regarding ethnicity, and genetic background and the small number of samples studied from each genotype group. However, few studies included HCV genotypes 1a and 3 to evaluate the impact of these mutations in insulin resistance. The absence of an association between mutations in HCV *core* aa 70 and 91 and the presence of IR suggests that genetic variability of this region has little impact on insulin resistance.

## 4. Materials and Methods

### 4.1. Study Population

Between 2011 and 2012, a total of 92 serum samples were collected from CHC patients residing in Rio de Janeiro that were referred to Viral Hepatitis Ambulatories in Rio de Janeiro (Oswaldo Cruz Institute, FIOCRUZ; Gaffree and Guinle University Hospital, UNIRIO and Clementino Fraga Filho University Hospital, UFRJ). Patients were included if they had CHC (anti-HCV and HCV-RNA positive for more than six months) and were 18 years of age and older. Exclusion criteria were: presence of diabetes type 1 (DT1) and 2 (DM2), hepatocellular carcinoma, human immunodeficiency virus (HIV) and/or hepatitis B co-infection, autoimmune liver disease, genetic liver disease (Wilson’s disease, hemochromatosis), previous HCV antiviral treatment, and excessive alcohol consumption. At the time in which blood samples were collected, no patients were treated. Some of them initiated antiviral therapy after the inclusion in the study. This study was approved by the institutional review boards. Informed consent was obtained from each patient who was included in the study, and the study protocol was followed according to the ethical guidelines of the 1975 Declaration of Helsinki (Ethical approval number: CAAE41269015.3.0000.5248).

### 4.2. Laboratorial Data

All serum samples were tested for anti-HCV using the commercial EIA kit HCV Ab (Radim, Pomezia, Italy), where all anti-HCV-reactive samples were retested in duplicate. HCV RNA viral load was determined using Abbott Real Time HCV m2000sp (Abbott Laboratories, Lake Bluff, IL, USA) and expressed as IU/mL.

A 12-h overnight fasting blood sample was drawn, to determine serum levels of alanine aminotransferase (ALT), γ-glutamyltransferase (GGT), total cholesterol, high-density lipoprotein (HDL) and low-density lipoprotein cholesterol (LDL), triglycerides, ferritin, plasma glucose concentration, and platelet count. Baseline serum vitamin D level was assessed by measuring serum 25-hydroxyvitamin D levels using automated immunochemiluminometric assay (ICMA) (Liason 25 (OH) Vitamin D, Diasorin, Varceli, Italy) with 20 ng/mL being considered the threshold value. Serum insulin was determined using ICMA (*imunoquimioluminescence*) (Liason Insulin Assay, Diasorin, Varceli, Italy). Insulin resistance (IR) was determined with the homeostasis model assessment method (HOMA) (IR: HOMA ≥ 2) (HOMA IR = fasting insulin (µUI/mL) × fasting glucose (mmol/L)/22.5). Hepatic fibrosis was assessed using Fib-4 and Forns index as the same as described previously [28]. 

### 4.3. Extraction of Viral RNA, Amplification of the Core Region by RT-PCR, and Sequence Analysis 

RNA was extracted from 140 µL of serum by QIAamp Viral RNA Mini Kit (Qiagen, Inc., Hilden, Germany) according to the manufacturer’s instructions. cDNA was synthesized by extension of random hexamers with Superscript III reverse transcriptase (Invitrogen, Carlsbad, CA, USA) [29]. 

The RT-PCR mixture contained 1 µL (10 µM) of the corresponding type of specific primers, 417 sense: 5′-GGYGGYGGNCAGATCGTTGG-3′ and 874 antisense: 5′-ARGAAGATAGARAARGAGCAACC-3′ 4 µL of dNTP (1.25 mM), 4 µL of PCR buffer (10×), 2 µL of MgCl_2_ (50 mM), 0.5 µL of Platinum^®^ Taq DNA Polymerase (5 U/µL), 7.5 µL of DNase/RNase free distilled water and 5 µL of cDNA. The conditions for the RT-PCR step were as follows: 94 °C for 4 min; then 40 cycles at 94 °C for 30 s, 55 °C for 60 s, 72 °C for 60 s, and a final elongation at 72 °C for 7 min. The RT-PCR conditions were the same for all genotypes. A total of 4 µL of the PCR1 product was submitted to a second round of PCR in the presence of 1 µL of each internal primer (10 µM) 439 sense: 5′-GAGTWTACBTGYTGCCGCGCAG-3′ and 1AS antisense: 5′-ATRTACCCCATGAGRTCGGC-3′, 2.5 µL of PCR buffer (10×), 4 µL of dNTPs (1.25 mM), 2.3 µL of MgCl_2_ (50 mM), 0.5 µL of Platinum^®^ Taq DNA Polymerase (5 U/µL), and 12.7 µL of DNase/RNase-free distilled water. After an initial denaturation at 94 °C for 2 min, the DNA was amplified for 40 cycles at 94 °C for 30 s, 59 °C for 45 s, and 72 °C for 60 s and subjected to a final extension at 72 °C for 7 min. PCR products of the expected length of 284 base pairs were fractionated by 1.5% agarose gel electrophoresis and stained with ethidium bromide.

PCR products were purified using the QIAquick gel extraction kit (Qiagen, Hilden, Germany) and submitted to nucleotide sequencing reactions in both directions using the Big Dye Terminator Cycle Sequencing Kit (version 3.1, Applied Biosystems, Foster City, CA, USA) according to the manufacturer’s instructions and analyzed on an ABI 3730 DNA automated sequencer (Applied Biosystems). The sequences obtained were aligned using the CLUSTALX program, version 1.83 and sequences were analyzed in MEGA v.6.0. After translation of nucleotide sequence into amino acids, protein sequences were evaluated in VESPA software to detect signatures or residues with different frequencies in the two sets of sequences (with and without IR). 

### 4.4. Statistical Analysis

Continuous data were expressed as mean ± SD, and the categorical data were expressed as numbers (percentages). Statistical differences in the categorical data were evaluated by the chi-squared test with Yates correction, or Fisher exact test. Distributions of continuous variables were analyzed by Student’s *t*-test or the Mann–Whitney *U* test for the two groups when appropriate. Multiple logistic regression analysis with stepwise variable selection was performed to assess the independent factors of higher HOMAIR ≥2. 

All evaluations were performed considering the genotype and subtype. Each group of samples with different genotypes and subtypes were evaluated separately considering their reference sequence, since differences in amino acids positions according to genotype are already known.

All the tests were performed with IBM SPSS Statistics 20.0 per Windows (Chicago, IL, USA).

## Figures and Tables

**Table 1:** Demographic, laboratory, and metabolic data of 92 CHC.

Variable	Value
Age (years)	54.84 (±10.88)
Gender	
Female	55 (59.8%)
Male	37 (40.2%)
25(OH)D ng/mL	27.82 (±12.29)
HCV RNA IU/mL	6.51 × 10^6^ (7.2 × 10^1^–3.06 × 10^8^)
Log_10_ HCV RNA IU/mL	5.81 (1.86–7.49)
Blood pressure	99.03 (±18.52)
Glucose ng/mL	92.84 (±13.51)
AST IU/mL	72.15 (±44.04)
ALT IU/mL	73.51 (±58.76)
VLDL mg/dL	18.96 (±7.80)
LDL mg/dL	105.500 (98–1069)
γGT IU/mL	94.25 (±77.66)
Insulin µU/mL	13.43 (±12.28)
HOMA	3.08 (±2.74)
TSH mIU/L	1.63 (0.07–11.49)
Hemoglobin g/dL	14.04 (±1.27)
Hematócrit (%)	41.65 (±3.60)
Phosphatase IU/mL	136.21 (±75.66)
Platelets10^3^/mm	187.13 (±72.72)
Sustained virological response (SVR)	
Without SVR	20 (21.7%)
With SVR	20 (21.7%)
No treatment	52 (56.5%)
Genotype	
1a	23 (25%)
1b	59 (64.1%)
3	10 (10.9%)
25(OH)D *	
<20	21 (22.8%)
≥20	70 (76.1%)
Homeostatic model assessment(HOMA)	
<2	52 (56.5%)
≥2	40 (43.5%)
Fibrosis algorithm	
Low	25 (54.3%)
High	67 (72.8%)

Continuous variables are expressed as mean value ± standard deviation. Percentage in parenthesis referred to the total of 92 patients. ALT: alanine aminotransferase. AST: aspartate aminotransferase. γGT: γ glutamyltransferase. LDL: low-density lipoprotein. * missing data: one patient.

**Table 2:** Univariate and multivariate regression analysis of factors associated with Insulin Resistance in 92 CHC patients.

Variable	Homeostatic Model Assessment (HOMA)		Bivariate Analysis *p* Value	Multivariate Analysis *p* (CI)
	<2	≥2		
Age (years)	52.42 (±11.49)	56.71 (±10.13)	0.061	−
Gender				
Female	32 (61.54%)	23 (57.5%)	0.695	−
Male	20 (38.46%)	17 (42.5%)		
25(OH)D ng/mL	28.43 (±10.92)	27.35 (±13.35)	0.681	−
HCV RNA IU/mL	6.51 × 10^6^ (7.2 × 10^1^–1.3× 10^7^)	2.13 × 10^6^ (1.34 × 10^2^–3.06 × 10^7^)	0.335	−
Log_10_ HCV RNA IU/mL	5.52 (±1.32)	5.26 (±1.47)	0.335	−
Glucose ng/mL	93.26 (±12.96)	92.61 (±14.04)	0.820	−
AST IU/mL	62.69 (±40.05)	79.43 (±45.93)	0.033	0.358 (0.989–1.030)
ALT IU/mL	71.02 (±57.35)	75.42 (±60.32)	0.795	−
VLDL mg/dL	19.33 (±8.85)	18.69 (±6.98)	0.785	−
LDL mg/dL	111.62 (28.7–996)	104.75 (40–155.40)	0.024	0.509 (0.975–1.013)
γGT IU/mL	78.44 (±71.07)	106.42 (±77.49)	0.024	0.217 (0.997–1.014)
TSH mIU/L	2.25 (±1.83)	1.86 (±1.55)	0.194	−
Hemoglobin g/dL	13.79 (±1.38)	14.23 (±1.16)	0.101	−
Hematocrit (%)	41.53 (±4.05)	41.75 (±3.26)	0.767	−
Phosphatase IU/mL	136.40 (±90.64)	136.07 (±77.17)	0.985	−
Platelets10^3^/mm	195.03 (±78.06)	181.07 (±68.48)	0.365	−
Sustained virological response (SVR)				
Without SVR	7 (17.5%)	13 (25%)	0.568	−
With SVR	8 (20%)	12 (23.08)		
No treatment	25 (62.50%)	27 (51.92)		
Genotype				
1a	9 (22.5%)	14 (26.92%)	0.839	−
1b	27 (67.5%)	32 (61.54%)		
3	4 (10%)	6 (11.54%)		
25(OH)D *				
<20	7 (17.50%)	14 (27.45%)	0.263	−
≥20	33 (82.5%)	37 (72.55%)		
Fibrosis algori thm				
Low	17 (42.5%)	8 (15.38%)	0.004	0.065 (0.106–1.070)
High	23 (57.50%)	44 (84.62%)		

Continuous variables were expressed as mean value ± standard deviation. Percentage in parenthesis referred to the total of 92 patients. ALT: alanine aminotransferase. AST: aspartate aminotransferase. γGT: γ glutamyltransferase. LDL: low- density lipoprotein. HOMA: homeostatic model assessments. * missing data: one patient.

**Table 3:** Evaluation of insulin resistance defined by HOMA index according genetic variability of HCV core gene (aa 70 and 91) in 92 patients with CHC.

Variable	Total N (%)	HOMA <2	≥2	Bivariate Analysis *p* Value
Any mutation in 70aa				
Wild type	50 (54.3%)	23 (57.5%)	27 (51.92%)	0.594
Mutant	42 (45.7%)	17 (42.5%)	25 (48.08%)	
70Q				
Wild type	63 (68.5%)	28 (70%)	35 (67.31%)	0.783
Mutant	29 (31.5%)	12 (30%)	17 (32.69%)	
70R				
Wild type	83 (90.2%)	37 (92.50%)	46 (88.46%)	0.518
Mutant	9 (9.8%)	3 (7.50%)	6 (11.54%)	
Any mutation in 91aa				
Wild type	78 (84.8%)	34 (85%)	44 (84.62%)	0.959
Mutant	14 (15.2%)	6 (15%)	8 (15.38%)	
Any mutation in 70 and 91aa				
Wild type	87 (94.6%)	39 (97.5%)	48 (92.31%)	0.276
Mutant	5 (5.4%)	1 (2.50%)	4 (7.69%)	

aa: amino acid. Genotype 3 wild type in aa 70 according reference sequence D49374.1 is P (proline). Genotype 1, subtypes 1a and 1b, in aa 70 according reference sequence NC_004102 and D90208, respectively, R (arginine). Genotype 3 wild type in aa91 according reference sequence D49374.1 is C (cysteine). Genotype 1, subtypes 1a and 1b, in aa91 according reference sequence NC_004102 and D90208, respectively, C (cysteine) and M (methionine).
